# A network analysis of nutritional markers and maternal perinatal mental health in the French EDEN cohort

**DOI:** 10.1186/s12884-023-05914-w

**Published:** 2023-08-23

**Authors:** Bethany Knox, Cédric Galera, Anne-Laure Sutter-Dallay, Barbara Heude, Blandine de Lauzon-Guillain, Judith van der Waerden

**Affiliations:** 1 Sorbonne Université, INSERM, Institut Pierre Louis d’Epidémiologie et de Santé Publique, Paris, F75012 France; 2 University of Bordeaux, Bordeaux, France; 3 INSERM, Bordeaux Population Health Center, UMR1219, Bordeaux, France; 4 Centre Hospitalier Perrens, Bordeaux, France; 5 Université Paris Cité and Université Sorbonne Paris Nord, INRAE, Centre for Research in Epidemiology and StatisticS (CRESS), Inserm, Paris, France; 6 Sorbonne Université, INSERM, Institut Pierre Louis d’Epidémiologie et de Santé Publique, Equipe de Recherche en Epidémiologie Sociale, 27 Rue Chaligny, Paris Cedex 12, 75571 France

**Keywords:** Perinatal mental health, Perinatal nutrition, Maternal health, Perinatal diet pattern, Network analysis

## Abstract

**Background:**

Perinatal maternal depression and anxiety are associated with adverse maternal outcomes, and nutrition may play an important role in their emergence. Previous research shows that certain micro and macronutrients found in different dietary patterns may associate with perinatal mood disorders. This study aims to explore relationships between nutrition during pregnancy and perinatal maternal depression and anxiety symptoms using network analyses.

**Methods:**

Using data from the French EDEN mother-child cohort, the sample consisted of 1438 women with available mental health outcomes (CES-D, STAI and EPDS) and nutritional markers collected from food frequency questionnaires. Four networks were constructed to explore the relationships between prenatal nutrient status, dietary patterns, and perinatal mental health, while accounting for important confounders.

**Results:**

The Healthy dietary pattern was associated with the presence of vital micronutrients, while the Western dietary pattern was consistently associated with poorer intake of specific micronutrients and contained an excess of certain macronutrients. Western dietary pattern and symptoms of postnatal depression were connected by a positive edge in both the macronutrient and micronutrient networks. Lower education levels were associated with higher Western dietary pattern scores, from which a positive edge linked to postnatal depression symptoms in both models.

**Conclusions:**

A Western dietary pattern was associated with increased symptoms of postnatal depression in our adjusted network models; The Healthy dietary pattern was associated with essential micronutrients but not with symptoms of depression or anxiety. Perinatal mental health might be impacted by specific dietary patterns in the context of psychosocial and physical stress associated with pregnancy.

**Supplementary Information:**

The online version contains supplementary material available at 10.1186/s12884-023-05914-w.

## Background

Perinatal depression and anxiety affect mothers worldwide and are associated with an increased likelihood of mental health problems in their offspring [1]. Prevalence rates during the perinatal period are estimated to be 11.9% for symptoms of depression and 22.9% for symptoms of anxiety [2, 3]. The need to identify factors that contribute to maternal mental illness, specifically during the vulnerable gestational and post-natal periods, remains a challenge. Historically, the literature has focused on common psychosocial risk factors including a personal or family history of mental health problems, adverse childhood events (ACE), or increased life stress [4, 5]. Nutrition has recently emerged as a factor that potentially contributes to perinatal anxiety and depression, possibly due to the increasing evidence of this association with mental health in the general population [6, 7]. Concomitantly, evidence links lower socioeconomic status (SES) and nutritional status, specifically a lack of micronutrients in the diets of lower SES individuals, creating a complex matrix of influential variables related to perinatal mental health [8].

The perinatal period is considered a time of intense nutritional demands for expectant mothers, mainly due to an increase in their resting metabolic rate [9]. Demanding pathophysiological changes require an additional intake of an extra 450 kcal/day on average throughout the course of pregnancy to account for total daily energy expenditure [9]. Throughout the life span, availability of vital nutrients is conditional upon patterns of dietary intake. Although no standardized definition of specific dietary patterns exists, previous studies have identified patterns such as ‘Healthy’ or ‘Western’ that generally consist of distinct food groups and their nutrients’ quality and quantity [10]. Research investigating dietary patterns and maternal mental health suggests that a ‘Healthy’ dietary pattern may have a protective effect against pre- and post-natal depression and potentially anxiety; however, the specific mechanisms surrounding these associations are not yet clear [11]. For example, a systemic review found a positive association between poor quality and unhealthy diets and prenatal depressive symptoms and that healthy diets were inversely associated with prenatal depressive and anxiety symptoms [12]. However, associations between perinatal diet and postnatal depressive remain inconsistent. Other previously studied factors increase the complexity of these mechanisms of action. This complexity is partly attributable to how nutrients are absorbed, processed, and utilized in the body during pregnancy and the ability to account for and accurately measure other factors that affect mental health. For example, Wang et al., found that in economically vulnerable women, a pro-inflammatory diet was associated with more prenatal depressive symptoms while the same association was not significant in women with a less-vulnerable economic status [13]. Research thus far has mainly focused on singular micronutrients or food groups with analyses restricted to dyadic correlations. This might fail to capture the complex interdependency between nutrients, the wider environment, and mental health. Additionally, many studies fail to adequately adjust for important confounders, including social determinants or history of mental health problems [14].

To address these issues, we propose the analytic framework of network analysis, which allows researchers to study dynamic systems of several mutually interacting components and their inter-relationships [15]. Often used in psychological research, this useful method enables the study of the complex interplay of variables whose nuance cannot be captured by traditional analytic procedures. Recently, network analysis has been proposed as a way to address “systems epidemiology” in which multiple risk factors can be simultaneously identified at a systems-level (e.g., clinical, biological, socio-economic) in place of the classic ‘one exposure’, ‘one outcome’ approach [16, 17]. Thus, our aim is not to produce an overall effect estimate but rather, visualize the inter-relationships between nutrition during pregnancy and maternal mental health, specifically the association between prenatal dietary patterns, nutrient intake, and the occurrence of symptoms of perinatal depression and anxiety in women in the French general population.

## Methods and materials

**Fig. 1:**
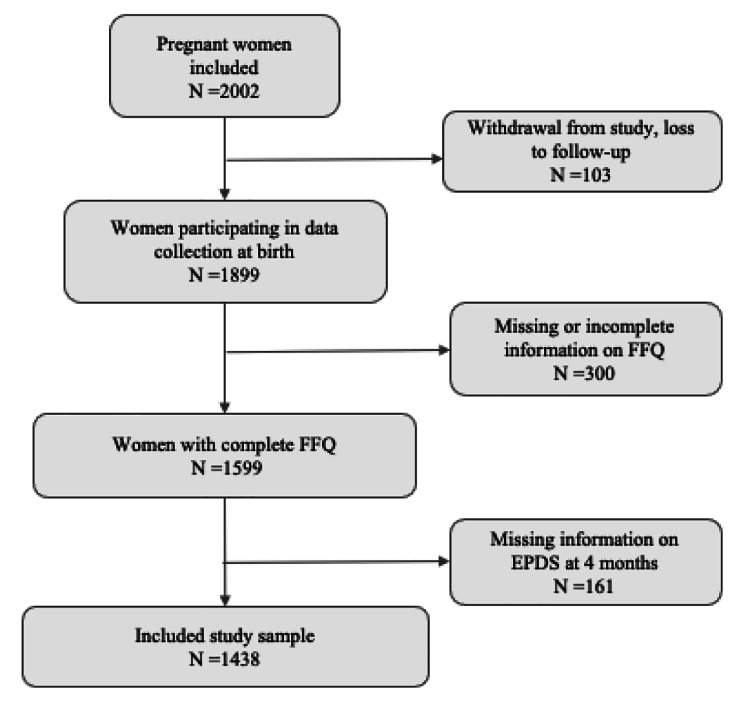
Study flowchart for EDEN cohort

Analysis was based on data from the French EDEN (Etude sur les déterminants pré et post natals précoces du Développement psychomoteur et de la santé de l’Enfant) mother-child cohort [18]. This cohort was set up to assess the pre- and postnatal nutritional, social, and environmental determinants of infant and child development and health. Participants were pregnant women recruited between 2003 and 2006 at two university maternity clinics in Nancy and Poitiers, France. Exclusion criteria were multiple pregnancies, a history of diabetes, intention to deliver outside the university hospital or move out of the study region within the next 3 years, and the inability to speak or read French. Informed consent was obtained from the mother at inclusion. Birth data were obtained from 1899 mother-infant pairs. During pregnancy and after birth, sociodemographic and biomedical data on the mother were gathered from obstetrical records, in face-to- face interviews with the mother, and by the mother’s self-completed questionnaires. Ethical approval for the collection and use of this data was approved by the ethics committee of Kremlin Bicêtre and by the Commission Nationale Informatique et Liberté. The current study sample consists of 1438 women with available data from food frequency questionnaires collected at birth, and mental health outcomes (see Fig. 1, flowchart).

### Perinatal maternal Mental Health

To assess the intensity of symptoms of prenatal depression, the Center for Epidemiological Studies-Depression Scale (CES-D) [19] was self-completed by the mother at inclusion between the 24th and 28th week of pregnancy. This 20-item questionnaire measures the intensity of depressive symptoms over the past week (score ranging 0–60) [20, 21] with higher scores indicative of increased intensity of symptoms.

The ‘state’ portion of the State-Trait Anxiety Inventory (STAI) [22] was used to assess symptoms of prenatal anxiety in mothers at the same data collection wave. This 20-question self-assessment (range 20–80) rates items on a 4-point scale with higher scores indicating greater anxiety.

Depressive symptoms at four months post-partum were assessed using the Edinburgh Postnatal Depression Scale (EDPS) a 10-item questionnaire (score ranging 0–30) designed to screen women at risk of postnatal depression [23]. All questionnaires have been translated and validated in French [20, 24, 25] with all scores being treated as continuous variables in the analysis.

### Prenatal maternal Nutrition

Maternal diet during the last trimester of pregnancy was assessed retrospectively during the mother’s stay at the maternity ward. Each participant completed a French validated Food Frequency Questionnaire (FFQ) that comprised 137 items with frequency ranges on a scale between ‘never’ and ‘more than once a day’ across seven categories. Portion sizes were determined using pictures for 12 food types (meat, French fries, pasta, vegetables, cakes, cheese etc.) on a three-level scale or were standard portions for the French adult population for other food types [26]. Each food item was assigned to one of the 44 food groups [27]. The nutrient composition database from the SU.VI.MAX study [26] was used to determine the nutrient content of foods and beverages. Nutrient values were only calculated for those women in the study who had two or fewer missing responses on the survey (n = 1599). The networks included the following *macronutrients*: total calories, vegetable protein, animal protein, saturated fats, monounsaturated fatty acids (MUFA), polyunsaturated fatty acids (PUFA), total cholesterol, carbohydrates, added sugars, and alcohol; and *micronutrients*: Vitamin A, Beta-carotene, Thiamin, Riboflavin, Niacin, Pantothenic Acid, Pyridoxine, Folic Acid, Cyanocobalamin, Ascorbic Acid, Vitamin D2 and D3, Tocopherol, Calcium, Iron, Iodine, Potassium, Magnesium, omega-3 fatty acid, and omega-6 fatty acid.

In a previous study, exploratory dietary patterns were derived by Principal Component Analysis (PCA, FACTOR procedure; SAS software) of the 44 standardized food groups consumed by more than half the sample [28]. The number of retained factors was determined by eigenvalues (the Scree plot) and based on their interpretability. A ‘Healthy’ dietary pattern (i.e., characterized by a high intake in fruits, vegetables, high fat dairy products, fish, legumes, and whole grains) and a ‘Western’ dietary pattern (i.e., characterized by a high intake of cakes, snacks, processed meat, prepared meals, soda, and chocolate) were identified. These two dietary patterns explained 10.8% (Western) and 6.8% (Healthy) variance of the first and second component of the PCA. A standardized score was calculated for each individual and pattern to quantify the individual’s adherence to the pattern. The current study uses these scores in our estimated networks as a continuous variable.

### Covariates

Information regarding covariates was collected between the 24th and 28th week of gestation via a face-to-face interview, examination, or medical record from the obstetrician’s office. Sociodemographic characteristics were age, migrant status, income, education level (years of formal education obtained), partner status, and parity. Covariates related to health include pre-pregnancy body mass index (BMI, kg/m^2^), gestational diabetes diagnosis, anemia diagnosis (pre-pregnancy), hypertension (pre-pregnancy), presence of nausea and vomiting, and vitamin supplementation during the 3rd trimester. Psychosocial covariates were Adverse Childhood Events (ACEs: yes vs. no), and history of mental health problems (yes vs. no).

### Statistical analysis

R (version 4.0.4) statistical software was used for data analysis. Descriptive analyses were performed on our sample (n = 1438) selected for network analysis based on exposure and outcome variable availability. Covariates included in the adjusted networks were determined based on subject knowledge and preliminary statistical testing using a p-value of 0.2. As the proportion of missing data was small (0.6%), all missing data points were removed. A complete case analysis was performed with the final data set as the missingness was determined to be missing completely at random [29, 30]. Preliminary data preparation and visualization analyses were run according to the guidelines provided by Epskamp et al. Networks were estimated using the R package *mgm* and plotted using the *qgraph* package which uses the Frutcherman-Reingold algorithm [31, 32]. Relevant patterns of interaction between variables can be visualized in a network structure in which variables are represented as ‘nodes’ while the presence of an *‘*edge’ between any 2 nodes represents a conditional dependence relation (red = negative, green = positive, grey = undefined) which accounts for all other nodes in the network. Thus, the presence of an edge indicates a statistical relation, however, does not specify the direction of the relation. The absence of an edge between two nodes signifies that they are conditionally independent and have no association within the network. The color saturation and width of an edge become darker and wider in proportion to the strength of relationship (weight) between variables. Sensitivity analysis (LASSO) was set to gamma of 0 and 0.25. Thus, we were able to see edges regardless of their capacity for shrinkage [33, 34]. We compared both models using the Pearson’s correlations test of the two pairwise weighted matrices and predictability estimates.

### Predictability, Node Centrality and Accuracy of Networks

We assessed the accuracy and stability of each network using centrality and predictability indices. Predictability refers to how well a node is predicted by its connected nodes [35] and is indicated by the colored perimeter of each node; centrality refers to the degree of connectivity a node has within the network [36]. These procedures inform the level of accuracy with which we can make inferences from our networks [37].

## Results

### Sample characteristics

Table 1 presents the characteristics of our 1438 study participants who responded to the EPDS survey at 4 months. When compared to the initial cohort, respondents tended to be slightly younger, more educated, have a higher income, were married or in a legal partnership, and experienced fewer adverse childhood events. There were no differences in the means of the prenatal depression survey scores, while prenatal anxiety scores were slightly lower in respondents. Over half of participants (61%) reported giving up certain foods during pregnancy and 15.6% reported being diagnosed with anemia prior to pregnancy. Many women reported a history of mental health problems (22.4%) and/or experiencing adverse childhood events (35.9%). In our sample, 23.8% of women reported clinical level symptoms of prenatal depression (CES-D score ≥ 16), 20.2% clinical level symptoms of prenatal anxiety (STAI ≥ 17), and 14.9% clinical level symptoms of post-natal depression (EPDS score ≥ 11).

**Table 1:** Participant characteristics non-respondents versus sample

	*Sample (n = 1438)*	Non-respondents (n = 564)*	
	*Mean (SD) or n (%)*	*Mean (SD) or n (%)*	*p-value*
***Demographics***			
*Age (years)*	29.6 (4.7)	29.0 (5.2)	0.028
*Migrant Status*			< 0.001
French	1237 (87.6%)	377 (66.8%)	
Descendent	135 (9.6%)	61 (10.8%)	
Immigrant	40 (2.8%)	37 (6.6%)	
*Income*			< 0.001
< 800€	46 (2.4%)	47 (8.3%)	
800–1500€	146 (7.6%)	88 (15.6%)	
1501–2300€	408 (21.3%)	160 (28.3%)	
2301–3000€	408 (21.3%)	93 (16.5%)	
3001–3800€	240 (12.5%)	54 (9.6%)	
>3800€	182 (9.5%)	41 (7.3%)	
*Education (years)*	13.90 (4.5)	12.64 (2.7)	< 0.001
*Partner Status*			< 0.001
Married/legal partnership	791 (55.2%)	216 (38.2%)	
Cohabitation with father	573 (40.0%)	216 (38.2%)	
Single/divorced/widow	69 (4.7%)	51 (9.0%)	
***Health Status***			
*BMI (*kg/m^2^)	23.17 (4.40)	23.49 (5.20)	0.189
*Gestational Diabetes Diagnosis*	90 (6.3%)	33 (5.9%)	0.613
*Anemia Diagnosis*	221 (15.4%)	78 (13.8%)	0.938
*Hypertension*	32 (2.2%)	5 (0.8%)	0.168
*Nausea and Vomiting*	1078 (55.5%)	412 (73.0%)	0.280
*Vitamin supplement 3rd trimester*	1045 (57.2%)	256 (45.4%)	< 0.001
*Parity*	0.78 (0.94)	0.91 (1.03)	
***Mental Health***			
*Adverse childhood event (ACE)*	515 (35.9%)	216 (38.2%)	0.001
*History of mental health problems*	323 (22.4%)	117 (20.7%)	0.517
*CES-D scores*	11.22 (7.6)	13.20 (9.16)	0.159
*STAI scores*	30.02 (9.33)	32.89 (11.04)	0.029
*EPDS scores*	5.29 (4.9)	NA	NA

*certain variable counts do not add up to total number of participants due to missing values

### Network structure

**Fig. 2:**
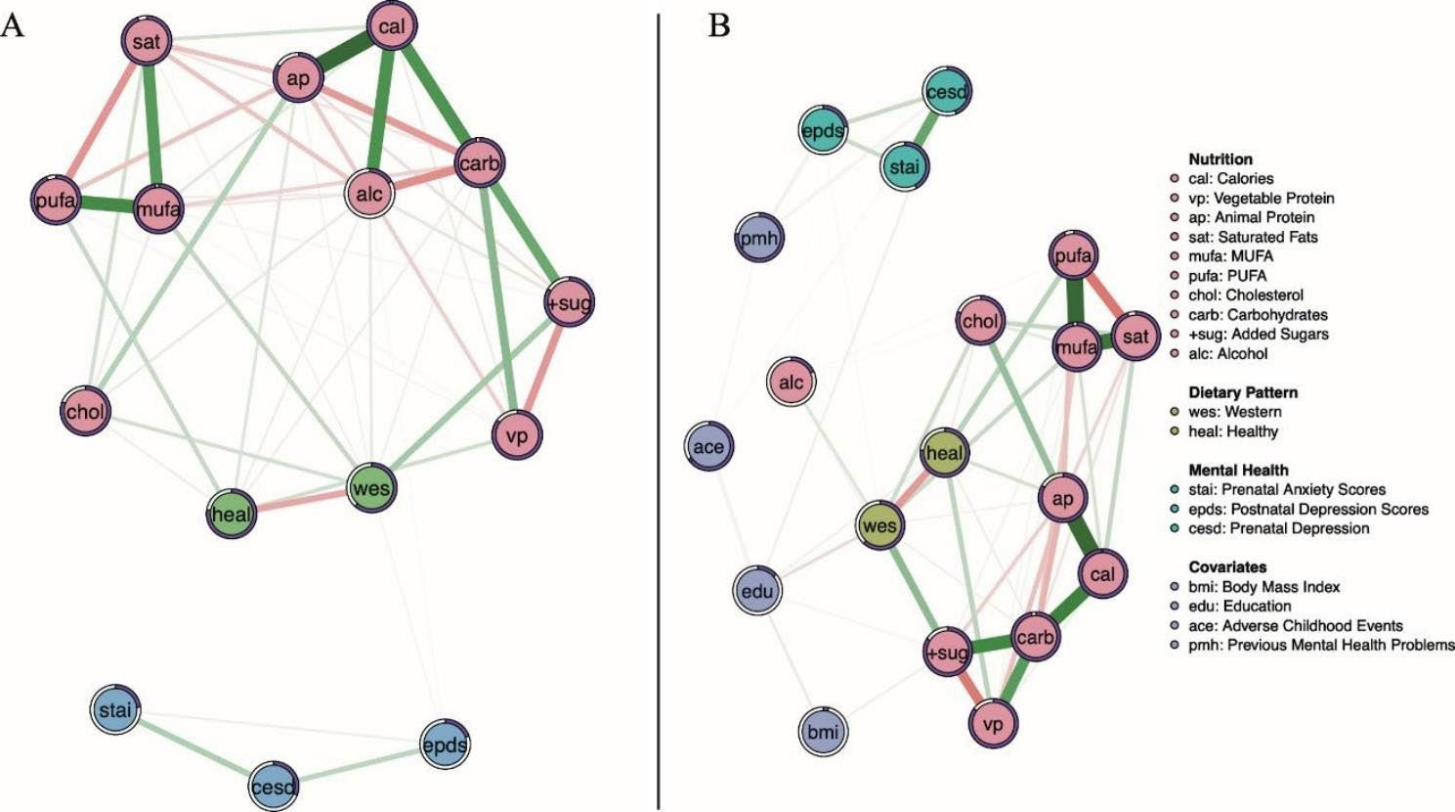
Network graph displaying the relationship between perinatal depression and anxiety scores symptoms and macronutrients before (panel **A**) and after controlling for covariates (panel **B**). Green edges signify positive partial correlations between variables, red edges signify negative partial correlations; shaded in dark purple rings around nodes relate the variance of that variable which represents to what extent it is explained by the nodes that connect to it

**Fig. 3:**
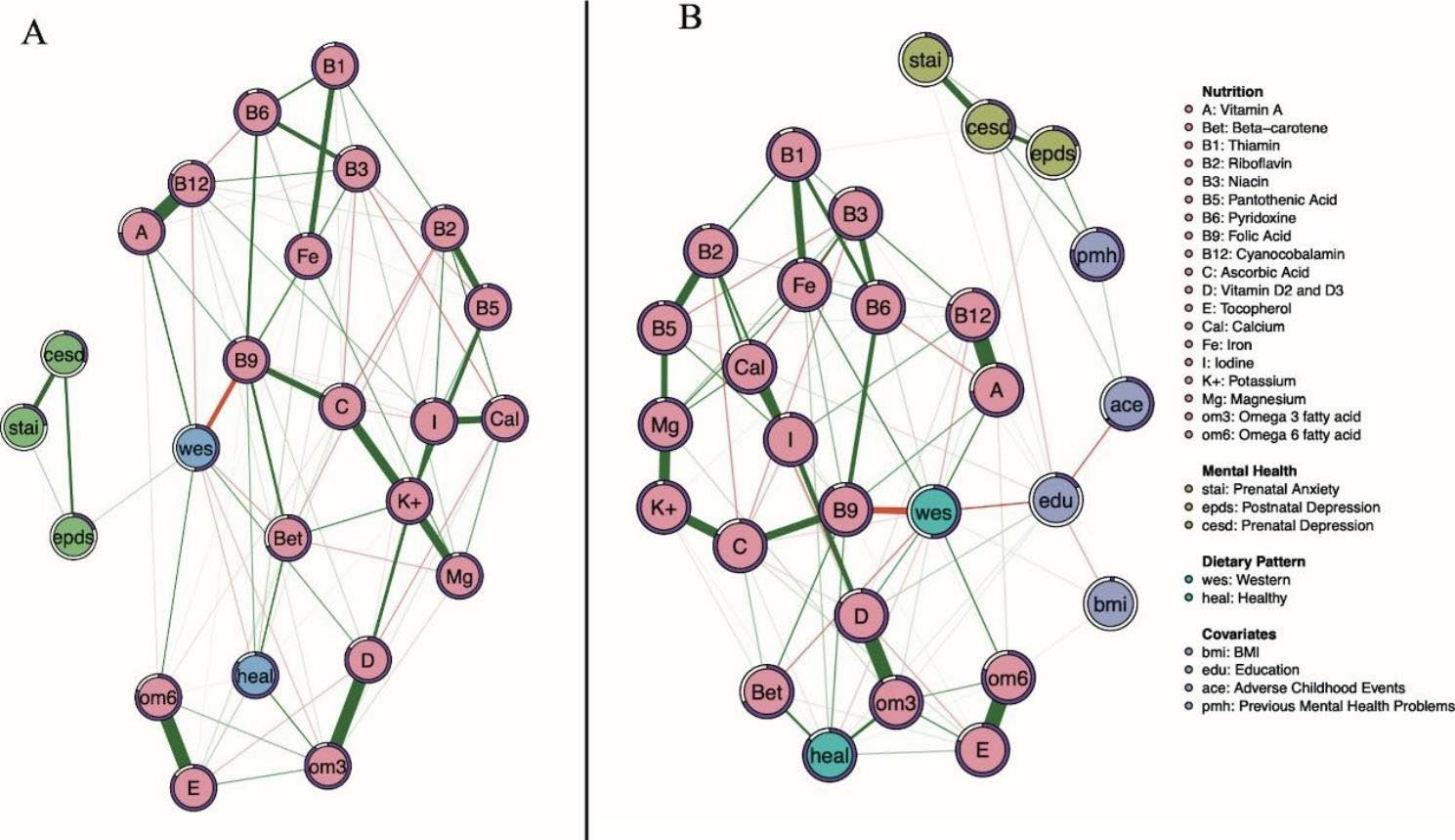
Network graph displaying the relationship between perinatal depression and anxiety scores symptoms and micronutrients before (panel **A**) and after controlling for covariates (panel **B**)

Figures 2 and 3 present the fully regularized models (LASSO) unadjusted and adjusted network graphs. We report edge weights (*r*) and predictability (*R*^*2*^) that are most relevant to the study. All relationships between nodes as described below are in reference to Panel B, the adjusted models.

The network graphs for the *Macronutrients* (Fig. 2) show a relationship between the Western dietary pattern and the EPDS score (*r* = 0.022). The prenatal CES-D and STAI scores are connected to the postnatal EPDS by positive, strong edges. The Western and Healthy dietary patterns have an inverse relationship (*r*= -0.359). Inverse relationships with the education node also include Western dietary pattern, CES-D, and BMI (*r*= -0.114, *r*= -0.052, *r*= -0.077). Other edges that connect to the Western dietary pattern are total cholesterol, monosaturated fatty acids, added sugars, and alcohol (*r* = 0.176, *r* = 0.187, *r* = 0.383, *r* = 0.116).

For the *Micronutrients* networks (Fig. 3), the positive relationship between the Western dietary pattern and EPDS scores (*r* = 0.025) also appears. Additionally, associations amongst covariates and the Western dietary pattern are displayed in a similar fashion to the macronutrient model (‘education’ connects to the Western diet (*r* = -0.21), CES-D (*r* = -0.07), adverse childhood events (*r* = -0.18), and BMI (*r* = -0.08)). As with the macronutrient model, pre and postnatal mental health scores are also connected by green edges. The Healthy dietary pattern appears to be positively associated with many essential micronutrients (B9, *r* = 0.15, Beta-carotene, *r* = 0.24 Omega-3, *r* = 0.25, Vitamin E, *r* = 0.08). The Western dietary pattern is positively associated with the essential nutrient Iron (*r* = 0.2), vitamin D (*r* = 0.16), Omega-6 (*r* = 0.2) and vitamin A (*r* = 0.21).

### Predictability, Node Centrality and Accuracy of Edges

**Fig. 4:**
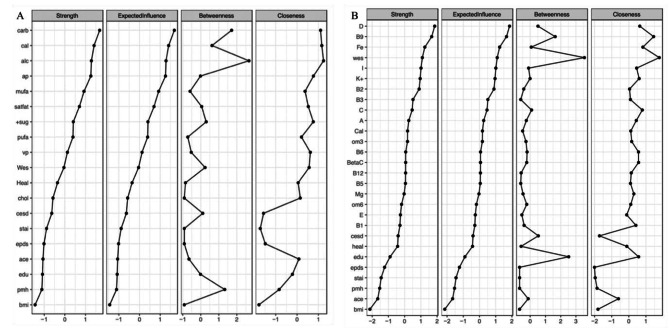
Node centrality measure of macronutrient (panel **A**) and micronutrient (panel **B**) network models. X-axis are z-scores

The Healthy diet had the highest predictability indicating that it is highly dependent upon essential micronutrients (R^2^ = 0.849, *Micronutrient adjusted*). Node centrality measures are presented in Fig. 4 (Panel A macronutrient adjusted model; Panel B micronutrient adjusted model respectively). Results from the parametric bootstrap demonstrate that edges were estimated reliably (Additional files 1 and 1b). Additionally, the ‘edge difference test’ indicates that there is a statistically significant difference between the values of any two edges in our networks (Additional files 2 and 2b).

## Discussion

To our knowledge, this is the first study to examine the relationship between prenatal nutrition and symptoms of depression and anxiety in the perinatal period using a network analysis approach. While previous literature has focused on either dietary patterns or isolated vitamins or minerals, we have elaborated on this research by identifying how both dietary patterns and their nutritional contents work in concert and may be associated with the intensity of perinatal depressive or anxious symptoms in the context of recognized risk factors. The intention of network analysis is not to present the traditional odds ratios, but rather to identify connections between a myriad of variables. In our networks, a Western dietary pattern was centrally located in both unadjusted and adjusted networks and either directly or indirectly connected with perinatal mental health and specific macro and micronutrients in a sample of women from the French general population. We found a small but positive association between postnatal depressive symptoms and prenatal Western dietary pattern score. Additionally, a Western dietary pattern and postnatal depressive symptoms were inversely linked by education. Contrary to previous findings [38, 39], we did not find associations between Healthy or Western diet patterns and symptoms of prenatal depression and anxiety or a relationship between postnatal depression scores and a Healthy dietary pattern. However, these results should be interpreted with caution, given the limitations of our study design and the possibility of reverse causality. History of mental health disorders and presence of prenatal mental health problems may likewise influence prenatal dietary choices, BMI, and a woman’s ability to adjust to the nutrient demands of pregnancy.

### Western versus healthy dietary patterns and Perinatal Mental Health

When exploring the additional role of dietary patterns and nutrients in perinatal depression and anxiety, their interplay warrants diligent detangling. Strategically, we used dietary pattern scores to reflect general food consumption, as well as measured nutrient content (grams/milligrams) in each food item to explore how they might relate to perinatal mental health. While direct associations between specific micro or macronutrients and the studied mental health outcomes do not appear, the Western dietary pattern was associated with postnatal depression after adjustment for other factors.

At the macronutrient level, the Western dietary pattern was related to higher intakes of monounsaturated fatty acids and added sugars. There is biological plausibility such macronutrient imbalances may disrupt proper functioning of neurological pathways. For example, overconsumption of monounsaturated fatty acids leads to changes in the phospholipid neuromembrane altering the rate of transmission of serotonin, dopamine, and norepinephrine, and possibly increasing risk for perinatal mood disorders [40]. Of note, the pathophysiological and psychosocial challenges surrounding pregnancy could make it difficult for a woman to change her dietary pattern at a time when it is most important, leading to an exacerbation of an pre-existing imbalances [41]. In the context of other risk factors, a Western diet and its components could aggravate an underlying risk to experience increased depressive symptoms, whereas a Healthy diet pattern would not. However, more human research concerning the consequences of overconsumption of certain macronutrients typically found in the Western dietary pattern as it relates to the etiology of perinatal depression and anxiety mood is needed.

At the micronutrient level, the Western dietary pattern was associated with lower intakes of phytochemicals such as folate, cobalamin, and beta-carotene, found to be associated with mood in the general population [6, 42]. Systematic reviews examining micronutrient deficiencies in pregnant women reveal that iron, zinc, and selenium are associated with an increased risk of post-partum depression [43, 44]. Although it is likely that most of our study participants were not nutritionally deficient in micronutrients, building evidence suggests that an *imbalance* of essential micronutrients during pregnancy can also affect the mother’s biochemistry at the cellular level [45]. Specifically for pregnant women, meta-analyses indicate that an imbalance of polyunsaturated fatty acids (n-3 versus n-6) could be affiliated with perinatal depression [46, 47]. While we saw no direct link in our networks between increased omega-6 and perinatal depression and anxiety, the postnatal depression node was linked to omega-6 via the Western diet node (stronger, positive edge weight), suggesting that the imbalance in omega fatty acids could be of importance in the context of those at risk for postnatal depression symptoms.

### Contributing factors

Finally, contrary to previous work, we have accounted for several contributing factors related to nutritional status and mental health. Previously studied risk factors of postnatal mood disorders appeared in our networks, namely prenatal symptoms of depression and anxiety, as well as mental health history. BMI is an important and well-studied confounder of diet patterns and mental health and risk of perinatal depression may be higher in women with increased BMI [48] Of note is that a prenatal Western dietary pattern was associated with a differentiated intake of certain nutrients and linked to BMI. An additional factor, education level, was associated with increased postnatal depression scores. Education level may reflect complexities regarding the ability to make appropriate choices about or access to nutrient-dense food. Higher education levels were previously associated with increased nutrient intake while lower income and educated groups were particularly lacking in micronutrients and diet quality. Moreover, it has been previously demonstrated that persons with a lower level of education or socioeconomic status experience a higher prevalence of mental health problems when compared to the general population [49]. Thus, it is possible that the consumption of a Western diet is one specific mechanism of action through which lower educational achievement, working in tandem with other biological and psychosocial risk factors, could contribute to postnatal depression.

### Strengths and Limitations

A strength of our study is its comprehensive approach in its attempt to disentangle the contribution of nutritional components in perinatal depressive and anxiety symptoms. Previous studies have tested one or few nutritional components in relation to perinatal depression and anxiety and have shown that certain macro- and micronutrients are beneficial. We used network analysis to identify patterns among these nutrients and related variables, and in addition, show that the Healthy dietary pattern is a correlate of the beneficial components which are lacking in a Western dietary pattern. We consider our study robust in that data from a large cohort gave our networks sufficient power. Moreover, this cohort is similar to other European mother-child dyad cohorts which could be used to easily replicate our study. Finally, our data analysis included the most conservative estimates and sensitivity testing of our networks so that only the most rigorous networks remained.

However, we should also acknowledge some limitations. First, both the dietary patterns and nutrient intakes were derived from a single time point food frequency questionnaire. We are limited in our lack of postnatal FFQs to verify the sustainability of the identified diet patterns and health behaviors that tend to fluctuate around the pregnancy period. Additionally, dietary patterns were assessed retrospectively at birth, introducing recall bias, and may not accurately measure nutrient intake during pregnancy. Although the FFQ has been validated, increasing confidence in the accuracy of its scores, biological samples of serum blood would be a more precise way to assess perinatal nutritional status at the micronutrient level and would allow to highlight nutrient deficiencies. Notwithstanding, it may take months before changes in dietary pattern are reflected in plasma levels of macro- and micronutrients, thus it is possible that the FFQ may be a more proximate measure to the time period in which our outcomes were assessed. Other methods to assess dietary intake, such as glycemic load, could lend further insights to the mechanism of action among these associations. For example, Wilson et al. found in a cohort of obese women that increased glycemic load was associated with a slight increase in depressive symptoms during the perinatal period [50]. Longitudinal cohort studies in which time-series data are collected would be needed to specify the evolution of nutritional status changes often associated with pregnancy and further clarify the exact role of dietary patterns in the context of perinatal mental health. Second, women’s mental health scores were self-reported and do not represent any official clinical evaluation of mental health status. It is important to highlight that the CES-D, STAI, and EPDS are not diagnostic tools and although used frequently in epidemiological studies, were created as screening devices to assess the presence of symptoms. However, all three scales have previously been validated in French and their continuous scores enabled us to study variations in symptomatology at the population level. Lastly, while network analysis allows for valuable global insights and pattern identification, it is statistically limited in its ability to make causal inferences and quantify risks. Our analysis uses the mixed graphical model which treats all variables equally and does not address temporality. In the instance that time-series data is available, more robust conclusions would be possible using the Vector Autoregressive Model to account for changes in nutrition status across time [31].

### Recommendations

Perinatal depression and anxiety are far-reaching public health issues that influence the health of mother, baby and the family unit. Improving dietary patterns and nutritional intake could be a safe, low-risk option to prevent or improve symptoms of depression and anxiety, and inherently has added benefits related to general health and wellness of the mother and development of the fetus. Nutritional intake is measurable, and deficits are easily recognized during preconception and in early pregnancy. Early discussion of dietary requirements and subsequent nutritional counseling is paramount in standard prenatal care. While very few nutritional counseling programs have been proven effective in the public sector, a recent systematic review assessed dietary intervention programs for pregnant women. It found that while effectiveness of the programs varied, almost half of the programs demonstrated an improvement in dietary behaviors and nutrition status of the women [51]. Nevertheless, more research is needed about the effectiveness of nutritional intervention programs and specifically, programs geared towards at-risk populations. In addition, whether improvement in nutritional intake during pregnancy leads to positive improvements with respect to maternal mental health status is still to be shown.

## Conclusion

The current study was the first to use network analysis to explore the associations between perinatal mental health and nutrition. Overall, our study provides additional evidence to support the theory that dietary pattern is related to the intake of essential micronutrients, and that a Western dietary pattern might adversely affect maternal postnatal mental health. As nutritional status is linked to dietary pattern, which is potentially influenced by education level, future studies should evaluate the effects of nutritional status on perinatal mental health within the context of other psychosocial risks.

### Electronic supplementary material

Below is the link to the electronic supplementary material.

**Supplement 1:** Bootstrapped difference test Macronutrient network model, adjusted. The grey area represents the bootstrapped confidence intervals, while the black line represents the sample values (edges). **Supplement 1b:** Bootstrapped difference test Micronutrient network model, adjusted.

**Supplement 2:** Edge-weight bootstrapped difference test, *Macronutrient* adjusted model. **Supplement 2b:** Edge-weight bootstrapped difference test, *Micronutrient* adjusted model.

## Data Availability

The data that support the findings of this study are available from the EDEN consortium but restrictions apply to the availability of these data, which were used under license for the current study, and so are not publicly available. Data are however available from the authors upon reasonable request and with permission of the EDEN consortium.

## Abbreviations

ACE: adverse childhood events
SES: socioeconomic status
EDEN: Etude sur les déterminants pré et post natals précoces du Développement psychomoteur et de la santé de l’Enfant
CES-D: Center for Epidemiological Studies-Depression Scale
STAI: State-Trait Anxiety Inventory
EDPS: Edinburgh Postnatal Depression Scale
FFQ: Food Frequency Questionnaire
SU.VI.MAX: Supplementation en Vitamines et Mineraux Antioxydants
MUFA: monounsaturated fatty acids
PUFA: polyunsaturated fatty acids
PCA: principal component analysis
BMI: body mass index
LASSO: least absolute shrinkage and selection operator
