# Supplementary material for: A network analysis of nutritional markers and maternal perinatal mental health in the French EDEN cohort
Source: BMC Pregnancy Childbirth. 2023 Aug 23;23:603. doi: 10.1186/s12884-023-05914-w (PMC10463670; doi:10.1186/s12884-023-05914-w)

A Network Analysis of Nutritional Markers and Maternal Perinatal Mental Health in the French EDEN Cohort

Online Resource 1: Bootstrapped difference test Macronutrient network model, adjusted. The grey area represents the bootstrapped confidence intervals, while the black line represents the sample values (edges).
Online Resource 1b: Bootstrapped difference test Micronutrient network model, adjusted

Online Resource 1: Bootstrapped difference test Micronutrient network model, adjusted

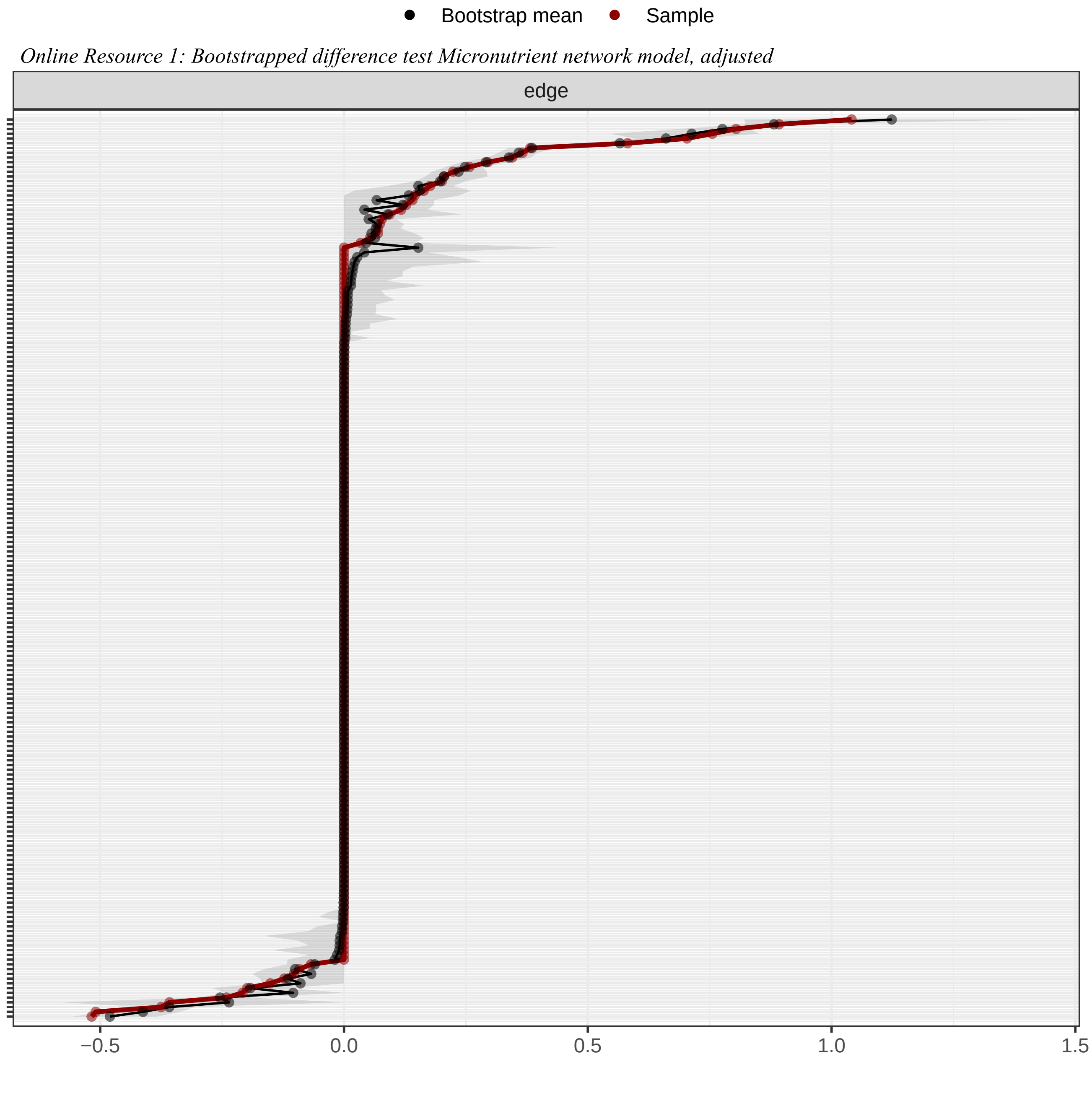

Online Resource 1b: Bootstrapped difference test Micronutrient network model, adjusted

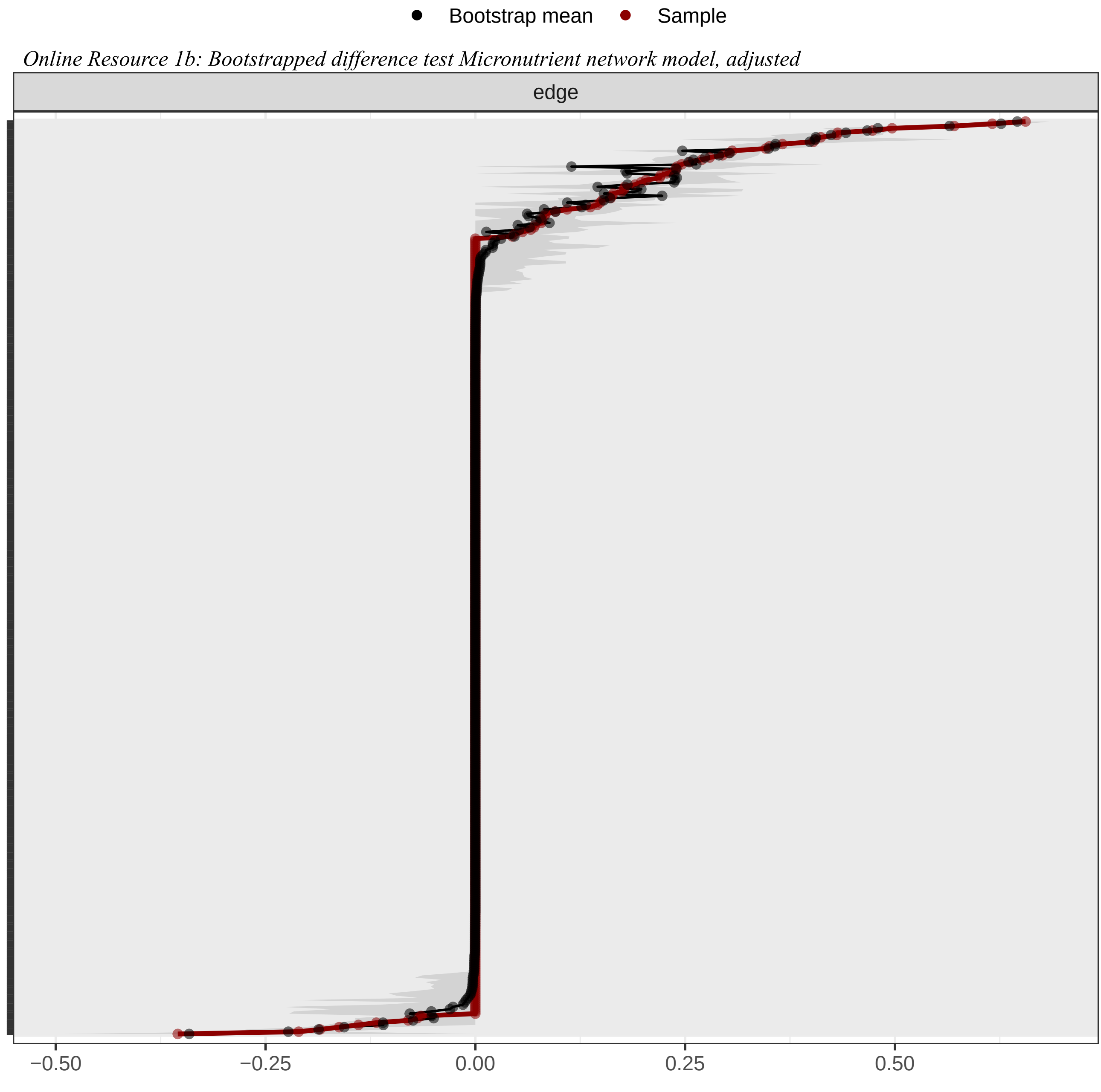

Supplement: Supplementary file 1 — Supplement 1: Bootstrapped difference test Macronutrient network model, adjusted. The grey area represents the bootstrapped confidence intervals, while the black line represents the sample values (edges). Supplement 1b: Bootstrapped difference test Micronutrient network model, adjusted. [file 12884_2023_5914_MOESM1_ESM.pdf]
