# Supplementary material for: A network analysis of nutritional markers and maternal perinatal mental health in the French EDEN cohort
Source: BMC Pregnancy Childbirth. 2023 Aug 23;23:603. doi: 10.1186/s12884-023-05914-w (PMC10463670; doi:10.1186/s12884-023-05914-w)
Supplement: Supplementary file 2 — Supplement 2: Edge-weight bootstrapped difference test, Macronutrient adjusted model. Supplement 2b: Edge-weight bootstrapped difference test, Micronutrient adjusted model. [file 12884_2023_5914_MOESM2_ESM.pdf]

A Network Analysis of Nutritional Markers and Maternal Perinatal Mental Health in the French EDEN Cohort

Online Resource 2: Edge-weight bootstrapped difference test, *Macronutrient* adjusted model. Black
Online Resource 2b: Edge-weight bootstrapped difference test, *Micronutrient* adjusted model

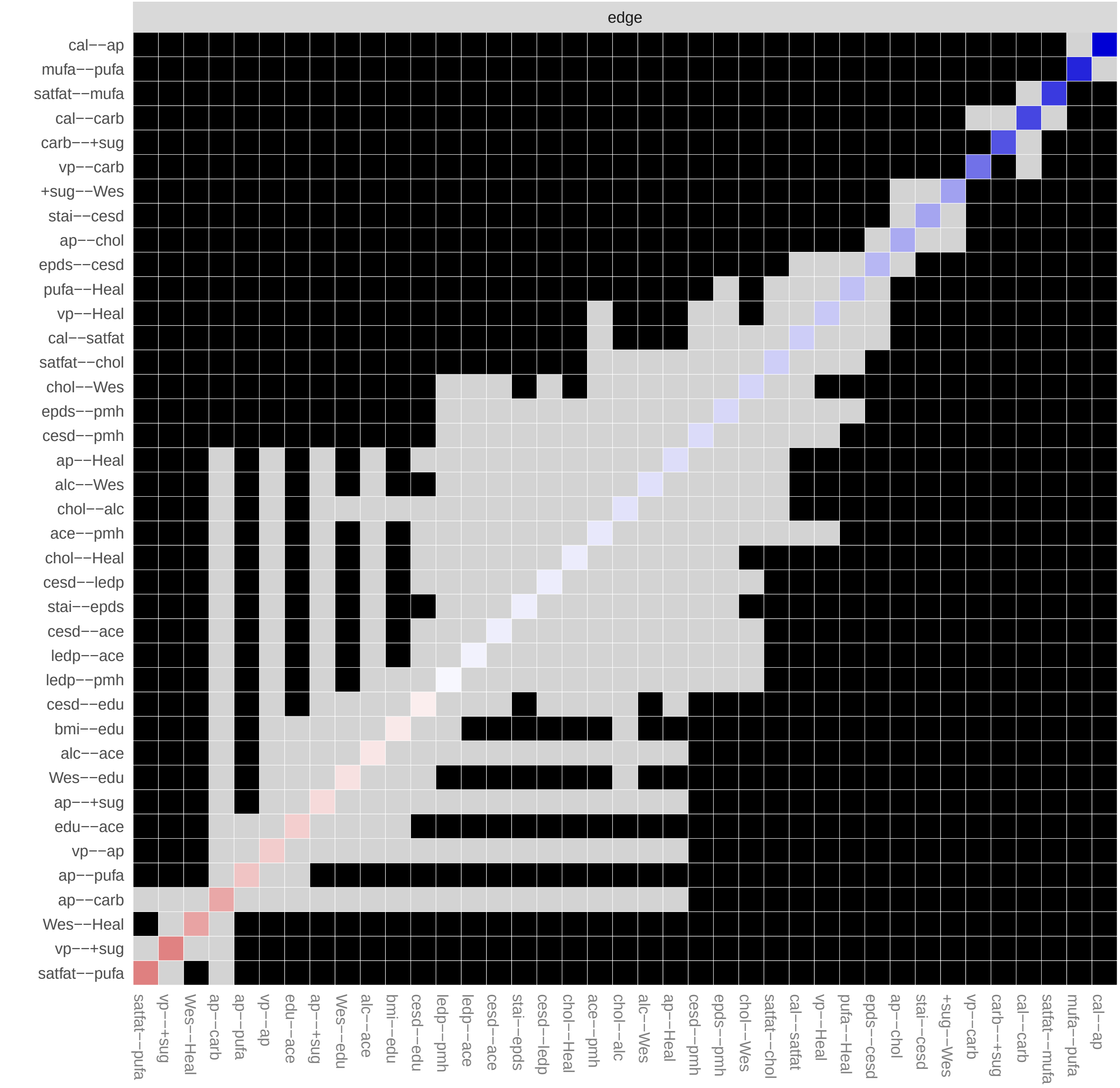

Online Resource 2: Edge difference tests for the edges in the estimated macronutrient network.

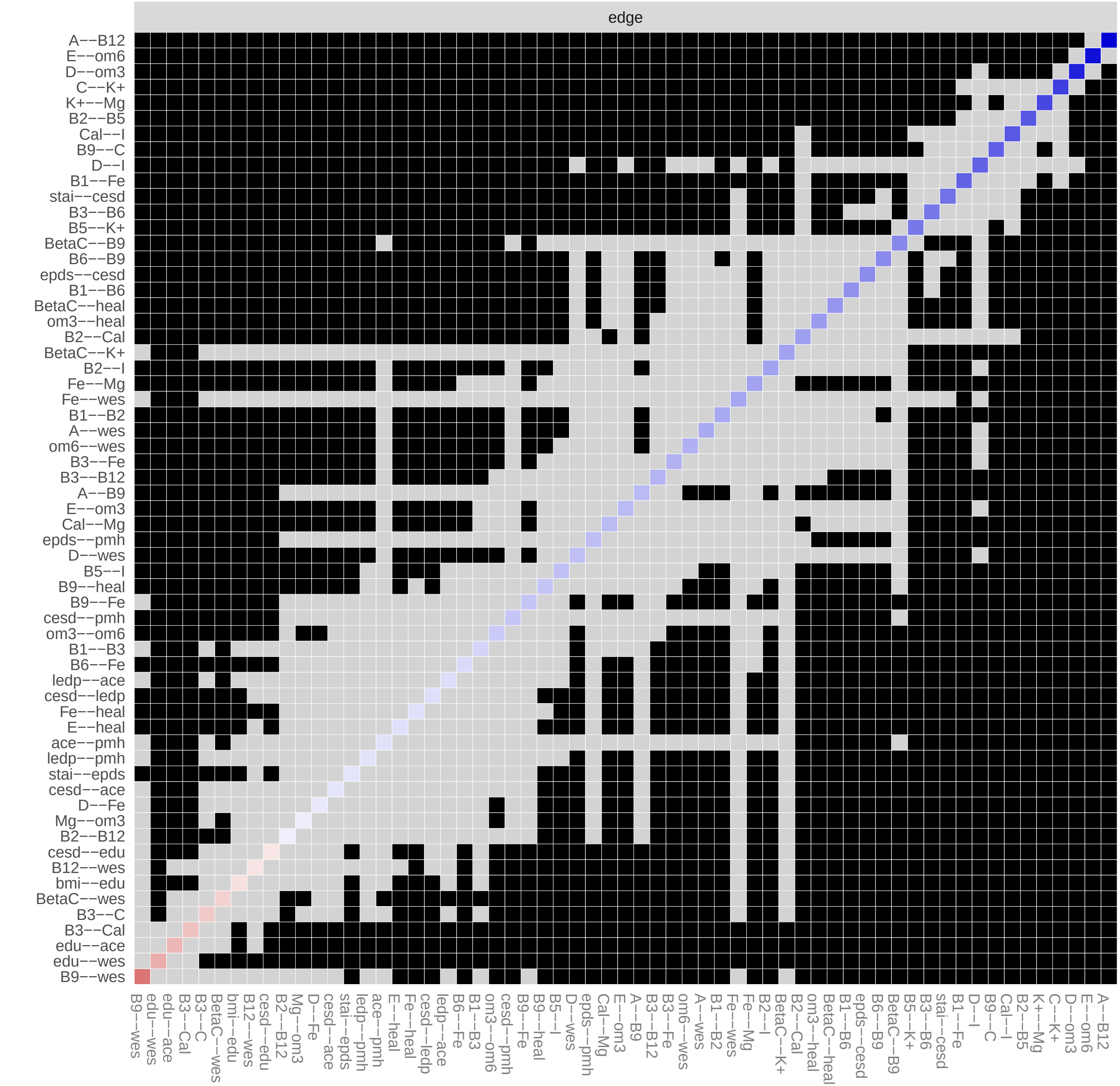

Online Resource 2b: Edge difference tests for the edges in the estimated micronutrient network.
